# Supplementary material for: Automatic Personalized Impression Generation for PET Reports Using Large Language Models
Source: ArXiv. 2023 Oct 17:arXiv:2309.10066v2. Preprint. [Version 2] (PMC10614982)
Supplement: 1 [file NIHPP2309.10066V2-supplement-1.pdf]

Supplementary Materials

Materials and Methods

Appendix S1: Statistics of PET Reports

Among 37,370 retrospective PET reports in our internal dataset, 92.7% (34,655/37,370) pertained to PET/CT whole-body (including skull base to thigh and skull vertex to feet) scans, 1.7% (649/37,370) to PET/MRI whole-body (including skull base to thigh and skull vertex to feet) scans, 5.5% (2,066/37,370) to PET limited area (including brain, cardiac and myocardial) scans. The findings section in a PET report had 346 [249, 472] (median [25th percentile, 75th percentile]) words, and the impression section had 86 [53, 130] words.

Appendix S2: "Description" and "Radiologist" Fields

In the input template, "Description" denotes the categories of PET scans, with their counts provided in Figure E1 (a). "Radiologist" accommodates a single token that encodes the reading physician's identity. The list of these tokens as well as their counts are given in Figure E1 (b). Notably, only physicians who dictated more than 100 PET reports are included.

Description	Counts
PET CT WHOLE BODY	34,655
PET CT BRAIN	1,424
PET MRI WHOLE BODY	649
PET CT MYOCARDIAL	407
PET MRI BRAIN	100
PET CT LIMITED AREA	91
PET MRI LIMITED AREA	29
PET CT CARDIAC	15

(a)

Tokens associated with dictating physicians	Counts	Tokens associated with dictating physicians	Counts	Tokens associated with dictating physicians	Counts
James	7184	Charles	827	Andrew	275
Robert	4872	Christopher	677	Kenneth	258
John	4827	Daniel	507	Kevin	241
Michael	4484	Matthew	460	Brian	178
David	3096	Anthony	408	George	173
William	2492	Mark	400	Timothy	157
Richard	1828	Donald	370	Ronald	156
Joseph	1231	Steven	358	Edward	154
Thomas	835	Paul	351	Jason	103

(b)

Figure E1: (a) shows the descriptions of examination categories in our internal dataset. (b) lists the reading physicians' unique identifier tokens.

Appendix S3: Models for PET Report Summarization

1. **PGN** (1) It is an encoder-decoder model built on the bidirectional long short-term memory (LSTM) architecture. The decoder can choose between copying a word directly from the input or generating a new one from the vocabulary. The model was modified to accommodate both background information and findings, as suggested in (1). We adapted the original implementation (available at github.com/yuhaozhang/summarize-radiology-findings) to fit our task and made the model weights accessible on GitHub: github.com/xtie97/PET-PGN.

2. **BERT2BERT** (2): It is an encoder-decoder model built on the transformer architecture. We utilized Clinical-Longformer (3) as the encoder and RoBERTa (4)as the decoder. The weights of the cross-attention layers were randomly initialized. Pretrained Clinical-Longformer is available on Hugging Face: huggingface.co/yikuan8/Clinical-Longformer and pretrained RoBERTa is available at huggingface.co/roberta-base.

3. **BART** (5): It is an encoder-decoder model built on the transformer architecture. BART introduced a denoising auto-encoder for pretraining, involving reconstructing the original texts from the corrupted samples. Pretrained BART is available at huggingface.co/facebook/bart-large.

4. **BioBART** (6): The model shares the same architecture with BART (5) but underwent further training on the PubMed dataset. Pretrained BioBART is available at huggingface.co/GanjinZero/biobart-large.

5. **PEGASUS** (7): It is an encoder-decoder model built on the transformer architecture. PEGASUS introduced a novel pretraining objective (gap sentence prediction), involving masking important sentences from documents and forcing the model to recover them based on the remaining sentences. Pretrained PEGASUS is available at huggingface.co/google/pegasus-large.

6. **T5** (8): It is an encoder-decoder model built on the transformer architecture. T5 established a unified framework that treats almost all natural language tasks as a text-to-text problem. Instead of the original T5, we used T5v1.1 that had multiple modifications of the architecture and was solely pretrained on unsupervised tasks. The model weights are available at huggingface.co/google/t5-v1_1-large.

7. **Clinical-T5** (9): It is tailored to handle the language structures, terminologies in medical documents by further pretraining T5 on the MIMIC-III dataset (10). The model weights are available at huggingface.co/luqh/ClinicalT5-large.

8. **FLAN-T5** (11): It is a variant of T5 that underwent instruction finetuning in a mixture of tasks. This enabled FLAN-T5 to achieve enhanced performance compared to the original T5 in various downstream applications. The model weights are available at huggingface.co/google/flan-t5-large.

9. **GPT2** (12): It is a decoder-only model built on the transformer architecture. Unlike the encoder-decoder models, GPT2 is pretrained on a massive corpus of text to predict the next word in a sequence. The model weights are available at huggingface.co/gpt2-xl.

10. **OPT** (13): It is a series of open-sourced, decoder-only transformers with varying sizes from 125M to 175B. The pretrained weights are available at huggingface.co/facebook/opt-1.3b.

11. **LLaMA-LoRA**: LLaMA (14) is a collection of decoder-only transformers, ranging from 7B to 65B. LLaMA-13B showed superior performance compared to GPT3 on most benchmarks. In this study, we chose LLaMA-7B and used LoRA (15) to accelerate training and reduce memory usage. The hyperparameters of the LoRA module are listed as follows: the rank of the low-rank factorization is 8, the scaling factor for the rank is 16, the dropout rate is 0.05, the target modules for LoRA are projection layers in query (q_proj) and value (v_proj). The model weights for LLaMA are available upon request.

12. **Alpaca-LoRA**: Alpaca (16) is the instruction tuned LLaMA-7B model that behaves qualitatively similarly to some closed-source large language models (LLMs), including OpenAI's text-davinci-003. When we finetuned Alpaca, we retained the same hyperparameters as used in LLaMA-LoRA. The weight difference between LLaMA and Alpaca is available at huggingface.co/tatsu-lab/alpaca-7b-wdiff.

All twelve language models were trained using the standard teacher-forcing algorithm. The training objective can be written as a maximum likelihood problem:

$$\theta^* = \underset{\theta}{\operatorname{argmax}} \sum_t \sum_i \log p_{G(\theta)} \left(r_t^{(i)} \middle| S^{(i)}, R_{<t}^{(i)}; \theta \right)$$

Where θ denotes the parameters of model G, $p_{G(\theta)}$ estimates the probability of the next word r_t given the previous sequence $R_{<t}$ in the reference text and the source text S. Superscript t denotes the word position in the reference text and i denotes a single sample. The AdamW optimizer (17) was employed

to optimize this log-likelihood loss. The learning rates for the transformer-based LLMs were selected from {5e-5, 1e-4, 2e-4, 4e-4} based on the Recall-Oriented Understudy for Gisting Evaluation-L (ROUGE-L) (18) in the validation set. We adopted the beam search decoding algorithm to generate impressions, setting the number of beams to 4. Additionally, we blocked the repeated trigram in the generated text and applied a length penalty of 2. For PGN, we followed the training and inference parameters specified in the original paper (1). Table E1 summarizes the settings for each model in this study.

The learning environment requires at least 2 NVIDIA A100 GPUs and the following Python (3.8.8) libraries: PyTorch (1.13.1), transformer (4.30.0), fastAI (2.7.11), deepspeed (0.9.2). Except for LLaMA-LoRA and Alpaca-LoRA, all models were trained on a single NVIDIA A100 GPU, with each epoch taking 50-120 minutes. LLaMA-LoRA and Alpaca-LoRA, however, required two NVIDIA A100 GPUs and took 4.5 hours per epoch.

Table E1: Training and inference settings of language models investigated in this study.

Language models	Finetuning methods	Number of trainable parameters	Learning rate	Total batch size	Number of training epochs	Number of beams for beam search
PGN	Full finetuning	8.3 M	1e-3 *	25 *	30 *	5 *
BERT2BERT	Full finetuning	301.7 M	1e-4	32	15	4
BART	Full finetuning	406.3 M	5e-5	32	15	4
BioBART	Full finetuning	406.3 M	5e-5	32	15	4
PEGASUS	Full finetuning	568.7 M	2e-4	32	15	4
T5	Full finetuning	783.2 M	4e-4	32	15	4
Clinical-T5	Full finetuning	737.7 M	4e-4	32	15	4
FLAN-T5	Full finetuning	783.2 M	4e-4	32	15	4
GPT2	Full finetuning	1.5 B	5e-5	32	15	4
OPT	Full finetuning	1.3 B	1e-4	32	15	4
LLaMA-LoRA	LoRA	4.2 M	2e-4	128	20	4
Alpaca-LoRA	LoRA	4.2 M	2e-4	128	20	4

Note that "*" denotes the hyperparameters directly taken from the original paper. Total batch size = training batch size per device × number of GPU devices × gradient accumulation steps.

Appendix S4: Benchmarking Evaluation Metrics

Both nuclear medicine (NM) physicians scored the quality of model-generated impressions on a 5-point Likert scale. The definition of each level are given in Table E2.

Table E2: Definition of the 5-point Likert scale for evaluating the quality of model-generated impressions.

Score	Definition
5	Clinically acceptable impressions. The generated impression is consistent with the key clinical findings and align with the physician's impression. Well organized and readable.
4	Nearly acceptable impressions. The generated impression is mostly consistent with the key clinical findings and aligns overall with the physician's impression. Minor additions or subtractions. Organized and readable.
3	Moderately acceptable impressions. The generated impression has some inconsistencies with the key clinical findings and mostly aligns with the physician's impression. Moderate additions or subtractions.
2	Unacceptable impressions. The generated impression is factually incorrect in parts and/or missing some key clinical findings and may not completely align with the physician's impression. Major additions or subtractions.
1	Unusable impressions. The generated impression is factually incorrect and/or misses most of the key clinically findings and does not align with the physician's impression.

We investigated a broad spectrum of evaluation metrics, comprising 17 different methods.

1. **ROUGE** (18): It measures the number of overlapping textual units between generated and reference texts. ROUGE-N (N=1,2,3) measures the overlap of N-grams, and ROUGE-L measures the overlap of longest common subsequence. ROUGE-LSUM extends ROUGE-L by computing the ROUGE-L for each sentence, and then summing them up.

2. **BLEU** (19): It computes the precision of n-gram overlap (n ranges from 1 to 4) between generated and reference texts with a brevity penalty.

3. **CHRF** (20): It computes the character-based n-gram overlap between the output sequence and the reference sequence. In this study, we set the n-gram length to 10.

4. **METEOR** (21): It computes an alignment of the generated text and the reference text based on synonymy, stemming, and exact word matching.

5. **CIDEr** (22): It computes the term frequency-inverse document frequency (TF-IDF) vectors for both human and machine-generated texts based on the n-gram (n ranges from 1 to 4) co-occurrence, and then measures the cosine similarity of the two vectors.

6. **ROUGE-WE** (23): It is an extension of the ROUGE metric, designed to assess the semantic similarity between generated and reference texts using pretrained word embeddings.

7. **BERTScore** (24): It evaluates the cosine similarity of contextual embeddings from BERT for each token in the output and reference sequences.

8. **MoverScore** (25): Similar to BERTScore, it leverages the power of BERT's contextual embeddings to measure the semantic similarity between generated and reference texts. Instead of token-level cosine similarity, MoverScore calculates the Earth Mover's Distance between the embeddings of the two texts.

9. **RadGraph** (26): It is a specialized evaluation metric tailored for radiology report summarization. RadGraph works by initially extracting clinical entities and their relations from the model-generated impression and the original clinical impression. Leveraging this data, it constructs knowledge graphs to compare the content coverage and structural coherence between the two impressions.

10. **BARTScore** (27): It leverages a pretrained BART model to compute the log probability of generating one text conditioned on another text. In this study, BARTScore is the BART model finetuned

on the CNN Daily Mail dataset. BARTScore+PET is the BART model finetuned on our internal PET report dataset. PEGASUSScore+PET is the PEGASUS model finetuned on our internal dataset. T5Score+PET is the FLAN-T5 model finetuned on our internal dataset. The training settings are the same as those in Table E1, except for different training/validation splits and random seeds.

11. **PRISM** (28): It is an evaluation metric used in multilingual machine translation. PRISM employs a sequence-to-sequence model to score the machine-generated output conditioned on the human reference.

12. **S^3** (29): It uses previously proposed evaluation metrics, including ROUGE and ROUGE-WE, as input features for a regression model to estimate the quality score of the generate text. S^3-resp is based on a model trained with human annotations following the responsiveness scheme, while S^3-pyr follows the pyramid scheme.

13. **UniEval** (30): It first constructs pseudo summaries by perturbing reference summaries, then defines evaluation dimensions using different prompt templates. The model is trained to differentiate pseudo data from reference data in a Boolean question-answering framework. While UniEval evaluates coherence, consistency, fluency, and relevance, we only present the overall score which is the average of these 4 dimensions.

14. **SummaQA** (31)It creates questions from the source document by masking entities. The generated text is then evaluated by a question-answering BERT model, with results reported in terms of the F1 overlap score.

15. **BLANC** (32): It measures how well a generated summary can help improve the performance of a pretrained BERT model in understanding each sentence from the source document with masked tokens.

16. **SUPERT** (33): It creates pseudo-reference summaries by extracting important sentences from the source document and then measures the semantic similarity between the generated text and this pseudo reference.

17. **Stats (Data Statistics)** (34): Stats-compression refers to the word ratio of the source document to its summary. Stats-coverage measures the proportion of words in the generated text that also appear in the source document. Stats-density is the average length of the fragment (e.g., sentence in the source document) from which each summary word is extracted. Stats-novel trigram is the percentage of trigrams present in the summary but absent in the source document.

For the metrics that have precision, recall and F1, we only present the F1 score, which is the harmonic mean of precision and recall. The evaluation codes are partially adapted from (35) and made available on GitHub: github.com/xtie97/PET-Report-Summarization/tree/main/evaluation_metrics.

Appendix S5: Implementation Details of Additional Analysis

1. **Deauville score (DS) extraction**: Whole-body PET reports that contained physician assigned DSs in the impression sections were identified by searching for the term "Deauville" and its common misspellings. N-gram analysis was then performed to extract the score for each case. Among 405 cases with DSs in the impression section, 34 cases also had DSs in the findings section. To avoid leakage, we removed the scores in these findings. If multiple DSs were present in the impression, the highest value was used to represent the exam-level DS (36). It is likely that model-generated impressions did not contain DSs in some cases, but their original clinical impressions had DSs or vice versa. Considering that we did not force the model to generate DSs in the impressions, we excluded these cases when

calculating 5-class accuracy and Cohen's κ index. Except for PGN, all language models had at least 250 cases available for evaluating the performance of DS prediction.

2. **Controlling reporting styles in output impressions**: To alter the style, we directly change the reading physician's identifier token to any option in Figure E1 (b). In this study, "Physician 1" corresponds to "Robert," "Physician 2" to "William," and "Physician 3" to "James". To illustrate, if we aim to generate the impression for a whole-body PET/CT report in the style of Physician 1, we need to replace the original reading physician's token with the token associated with Physician 1 (i.e., "Robert"). For encoder-decoder models, the input should start with "Description: PET CT WHOLE BODY Radiologist: Robert". For decoder-only models, the instruction should be "Instruction: Derive the impression from the given PET CT WHOLE BODY report for Robert".

Results

Appendix S6: Correlation of Evaluation Metrics with the Second Physician's Scores

Figure E2 presents the Spearman's ρ correlation between evaluation metrics and quality scores assigned by the second physician (S.Y.C.). BARTScore+PET and PEGASUSScore+PET showed the highest correlation values. Both physicians agreed upon the top-5 metrics most correlated with physician preferences, namely BARTScore+PET, PEGASUSScore+PET, T5Score+PET, UniEval and BARTScore.

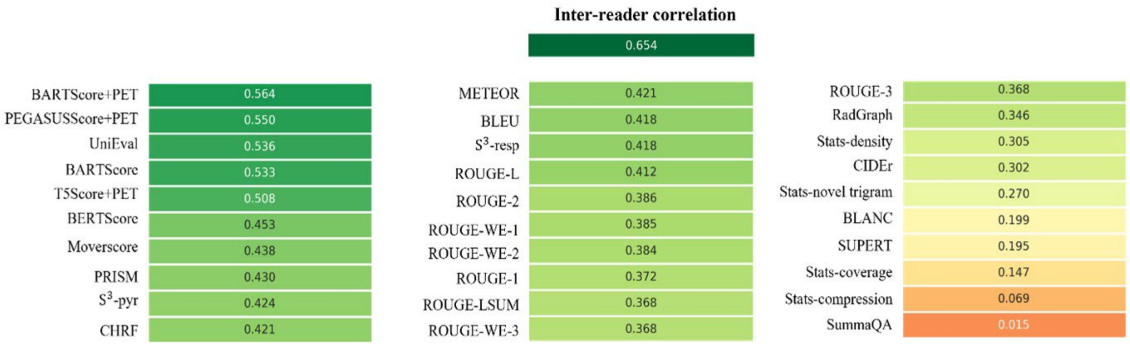

Figure E2: Spearman's ρ correlations between different evaluation metrics and quality scores assigned by the second physician.

Appendix S7: Model Performance

Figure E3 presents the performance evaluation of 12 language models across all 30 metrics (17 different methods) considered in this study. All numbers in this figure are actual metric values. In the first column, we sort the metrics in descending order of correlation with the first physician's (M.S.) preference.

	PGN	BERT2 BERT	BART	BioBART	PEGASUS	T5	Clinical-T5	FLAN-T5	GPT2	OPT	LLaMA-LoRA	Alpaca-LoRA
BARTScore +PET	-2.25 [-2.26, -2.23]	-1.61 [-1.63, -1.60]	**-1.46 * [-1.47, -1.44]**	**-1.46 † [-1.47, -1.45]**	**-1.47 † [-1.48, -1.46]**	-1.53 [-1.54, -1.51]	-1.54 [-1.56, -1.53]	-1.54 [-1.56, -1.53]	-2.04 [-2.05, -2.03]	-2.07 [-2.08, -2.05]	-2.27 [-2.28, -2.25]	-2.24 [-2.25, -2.22]
PEGASUSScore +PET	-2.25 [-2.27, -2.23]	-1.55 [-1.56, -1.53]	-1.49 [-1.50, -1.47]	-1.48 [-1.49, -1.47]	**-1.44 * [-1.45, -1.42]**	-1.46 [-1.47, -1.45]	-1.50 [-1.51, -1.48]	-1.48 [-1.49, -1.46]	-2.26 [-2.28, -2.24]	-2.27 [-2.28, -2.25]	-2.48 [-2.50, -2.46]	-2.46 [-2.47, -2.44]
T5Score+PET	-2.20 [-2.22, -2.19]	-1.52 [-1.53, -1.50]	-1.46 [-1.47, -1.44]	-1.44 [-1.46, -1.43]	**-1.42 † [-1.43, -1.40]**	**-1.41 * [-1.42, -1.39]**	-1.45 [-1.46, -1.43]	**-1.42 † [-1.44, -1.41]**	-2.17 [-2.19, -2.16]	-2.20 [-2.21, -2.18]	-2.38 [-2.40, -2.36]	-2.36 [-2.38, -2.34]
UniEval	0.34 [0.34, 0.35]	0.72 [0.71, 0.72]	0.76 [0.75, 0.76]	0.76 [0.76, 0.77]	**0.78 * [0.78, 0.78]**	0.77 [0.77, 0.78]	0.77 [0.77, 0.77]	0.78 [0.77, 0.78]	0.64 [0.63, 0.64]	0.59 [0.59, 0.60]	0.68 [0.68, 0.69]	0.68 [0.67, 0.68]
BARTScore	-3.97 [-3.99, -3.95]	-3.20 [-3.22, -3.18]	**-3.06 † [-3.08, -3.04]**	**-3.07 † [-3.09, -3.05]**	**-3.05 * [-3.07, -3.03]**	**-3.07 † [-3.09, -3.05]**	-3.10 [-3.12, -3.08]	**-3.06 † [-3.08, -3.04]**	-3.81 [-3.83, -3.80]	-3.82 [-3.83, -3.80]	-3.93 [-3.95, -3.92]	-3.93 [-3.94, -3.91]
CHRF	25.3 [24.9, 25.6]	36.3 [35.9, 36.7]	40.9 [40.5, 41.3]	40.0 [39.6, 40.4]	**42.0 † [41.6, 42.4]**	41.1 [40.7, 41.5]	41.1 [40.7, 41.5]	**42.2 * [41.8, 42.6]**	29.2 [28.9, 29.6]	31.6 [31.3, 31.9]	25.7 [25.4, 26.0]	26.0 [25.7, 26.3]
Moverscore	0.565 [0.563, 0.568]	0.592 [0.590, 0.594]	0.601 [0.599, 0.603]	0.602 [0.600, 0.604]	**0.607 † [0.605, 0.608]**	**0.607 † [0.605, 0.608]**	0.605 [0.604, 0.607]	**0.607 * [0.606, 0.609]**	0.575 [0.574, 0.576]	0.576 [0.575, 0.577]	0.570 [0.569, 0.570]	0.572 [0.571, 0.573]
BLEU	10.8 [10.5, 11.1]	18.7 [18.3, 19.1]	22.6 [22.2, 23.1]	22.5 [22.1, 22.9]	**24.7 † [24.2, 25.1]**	24.1 [23.7, 24.6]	23.9 [23.5, 24.4]	**24.7 * [24.3, 25.2]**	11.4 [11.1, 11.6]	11.7 [11.4, 11.9]	9.3 [9.1, 9.6]	9.6 [9.4, 9.9]
BERTscore	0.673 [0.735, 0.739]	0.723 [0.735, 0.739]	0.735 [0.735, 0.739]	0.737 [0.735, 0.739]	0.744 [0.735, 0.739]	**0.747 * [0.735, 0.739]**	0.743 [0.735, 0.739]	**0.747 † [0.735, 0.739]**	0.685 [0.735, 0.739]	0.683 [0.735, 0.739]	0.673 [0.735, 0.739]	0.677 [0.735, 0.739]
ROUGE-WE-1	38.9 [38.4, 39.3]	49.2 [48.8, 49.6]	52.5 [52.0, 52.9]	52.3 [51.9, 52.8]	**54.4 † [54.0, 54.8]**	**54.4 † [54.0, 54.8]**	54.0 [53.6, 54.4]	**54.8 * [54.4, 55.2]**	42.2 [41.8, 42.5]	43.2 [42.8, 43.5]	38.1 [37.8, 38.4]	38.9 [38.6, 39.3]
ROUGE-1	37.8 [37.4, 38.2]	48.4 [48.0, 48.7]	51.9 [51.5, 52.4]	51.8 [51.3, 52.2]	**53.8 † [53.4, 54.2]**	**53.7 † [53.3, 54.1]**	53.2 [52.8, 53.6]	**54.1 * [53.7, 54.5]**	41.6 [41.3, 42.0]	42.6 [42.2, 42.9]	38.4 [38.1, 38.8]	39.2 [38.8, 39.6]
ROUGE-L	28.7 [28.3, 29.1]	35.9 [35.5, 36.4]	38.6 [38.1, 39.1]	38.9 [38.4, 39.4]	**40.0 † [39.6, 40.5]**	**40.3 * [39.9, 40.8]**	39.4 [39.0, 39.9]	**40.2 † [39.7, 40.7]**	28.7 [28.4, 29.1]	28.3 [27.9, 28.7]	27.2 [26.9, 27.6]	28.0 [27.6, 28.3]
ROUGE-LSUM	35.4 [34.9, 35.8]	45.1 [44.7, 45.5]	48.7 [48.2, 49.1]	48.6 [48.2, 49.1]	**50.5 † [50.0, 50.9]**	**50.4 † [49.9, 50.8]**	49.8 [49.4, 50.2]	**50.8 * [50.4, 51.2]**	38.3 [38.0, 38.7]	39.2 [38.9, 39.6]	35.4 [35.0, 35.7]	36.0 [35.7, 36.4]
ROUGE-WE-2	25.6 [25.2, 26.0]	35.6 [35.2, 36.0]	38.8 [38.4, 39.3]	38.6 [38.1, 39.0]	**40.3 † [39.8, 40.7]**	**40.2 † [39.8, 40.7]**	39.9 [39.4, 40.3]	**40.7 * [40.2, 41.1]**	26.8 [26.4, 27.1]	27.6 [27.2, 27.9]	22.7 [22.4, 23.0]	23.5 [23.2, 23.9]
METEOR	0.180 [0.177, 0.182]	0.232 [0.229, 0.235]	0.267 [0.264, 0.270]	0.262 [0.259, 0.265]	**0.276 * [0.273, 0.279]**	0.272 [0.269, 0.275]	0.272 [0.269, 0.275]	**0.279 † [0.276, 0.281]**	0.195 [0.192, 0.197]	0.213 [0.211, 0.215]	0.169 [0.167, 0.171]	0.172 [0.170, 0.174]
ROUGE-WE-3	26.5 [26.1, 26.9]	37.2 [36.8, 37.7]	40.8 [40.3, 41.3]	40.5 [40.0, 41.0]	**42.3 † [41.8, 42.7]**	**42.1 † [41.6, 42.5]**	41.6 [41.1, 42.0]	**42.5 * [42.0, 43.0]**	28.3 [27.9, 28.7]	29.4 [29.1, 29.8]	22.9 [22.5, 23.2]	24.0 [23.6, 24.4]
RadGraph	0.225 [0.221, 0.230]	0.348 [0.343, 0.352]	0.381 [0.376, 0.386]	0.383 [0.378, 0.388]	**0.395 † [0.390, 0.400]**	0.388 [0.383, 0.393]	**0.393 † [0.388, 0.398]**	**0.397 * [0.392, 0.402]**	0.221 [0.217, 0.225]	0.235 [0.232, 0.239]	0.177 [0.174, 0.180]	0.190 [0.186, 0.193]
ROUGE-2	17.9 [17.5, 18.3]	26.3 [25.9, 26.8]	29.6 [29.1, 30.0]	29.4 [29.0, 29.9]	**30.9 * [30.5, 31.4]**	**30.7 † [30.2, 31.1]**	30.1 [29.6, 30.5]	**30.9 † [30.4, 31.4]**	15.9 [15.6, 16.2]	16.1 [15.8, 16.4]	13.4 [13.1, 13.6]	13.9 [13.6, 14.2]
PRISM	-3.96 [-3.98, -3.94]	-3.40 [-3.42, -3.37]	-3.34 [-3.37, -3.32]	-3.29 [-3.32, -3.27]	**-3.26 † [-3.28, -3.24]**	**-3.24 * [-3.26, -3.22]**	-3.29 [-3.31, -3.26]	**-3.26 † [-3.28, -3.24]**	-3.99 [-4.01, -3.97]	-4.02 [-4.05, -4.00]	-4.07 [-4.09, -4.05]	-4.07 [-4.09, -4.05]
ROUGE-3	10.3 [10.0, 10.7]	16.5 [16.1, 17.0]	19.3 [18.9, 19.8]	19.4 [18.9, 19.8]	**20.5 * [20.1, 21.0]**	**20.2 † [19.7, 20.6]**	19.7 [19.3, 20.2]	**20.4 † [19.9, 20.8]**	6.8 [6.5, 7.1]	6.7 [6.5, 7.0]	5.2 [5.0, 5.4]	5.5 [5.3, 5.7]
S^3-pyr	0.37 [0.37, 0.38]	0.58 [0.57, 0.58]	**0.70 † [0.69, 0.71]**	0.66 [0.65, 0.67]	**0.70 † [0.69, 0.71]**	0.68 [0.67, 0.69]	0.68 [0.67, 0.69]	**0.71 * [0.70, 0.71]**	0.44 [0.43, 0.45]	0.52 [0.51, 0.52]	0.36 [0.35, 0.36]	0.37 [0.36, 0.37]
S^3-resp	0.51 [0.50, 0.52]	0.67 [0.67, 0.68]	**0.78 † [0.77, 0.79]**	0.75 [0.74, 0.76]	**0.78 † [0.77, 0.79]**	0.77 [0.76, 0.77]	0.76 [0.76, 0.77]	**0.79 * [0.78, 0.79]**	0.53 [0.53, 0.54]	0.58 [0.58, 0.59]	0.48 [0.47, 0.48]	0.49 [0.48, 0.49]
Stats-novel trigram	0.85 [0.84, 0.85]	0.76 [0.76, 0.77]	0.68 [0.68, 0.69]	0.69 [0.68, 0.69]	0.62 [0.61, 0.62]	0.68 [0.68, 0.69]	0.65 [0.64, 0.65]	0.65 [0.65, 0.66]	0.98 [0.98, 0.98]	**0.99 † [0.99, 0.99]**	**0.99 * [0.99, 0.99]**	**0.99 † [0.99, 0.99]**
Stats-density	1.89 [1.85, 1.92]	2.98 [2.92, 3.04]	5.43 [5.27, 5.59]	5.49 [5.32, 5.66]	**6.51 * [6.34, 6.68]**	4.64 [4.53, 4.76]	5.45 [5.31, 5.58]	5.47 [5.33, 5.61]	0.87 [0.86, 0.88]	0.85 [0.85, 0.86]	0.77 [0.77, 0.78]	0.78 [0.77, 0.79]
CIDEr	0.179 [0.159, 0.199]	0.445 [0.411, 0.479]	0.556 [0.517, 0.594]	0.546 [0.507, 0.584]	**0.637 * [0.597, 0.677]**	**0.599 † [0.560, 0.639]**	**0.600 † [0.561, 0.640]**	**0.631 † [0.591, 0.671]**	0.184 [0.166, 0.202]	0.203 [0.182, 0.224]	0.125 [0.113, 0.137]	0.152 [0.136, 0.167]
BLANC	0.049 [0.047, 0.051]	0.089 [0.086, 0.091]	0.122 [0.119, 0.124]	0.113 [0.111, 0.116]	**0.131 * [0.128, 0.134]**	0.114 [0.112, 0.117]	0.126 [0.123, 0.128]	0.126 [0.123, 0.129]	0.053 [0.051, 0.054]	0.061 [0.059, 0.063]	0.045 [0.043, 0.047]	0.044 [0.042, 0.046]
Stats-compression	**8.36 * [8.20, 8.52]**	6.16 [6.04, 6.28]	5.31 [5.18, 5.44]	5.51 [5.40, 5.62]	5.49 [5.37, 5.61]	5.78 [5.66, 5.90]	5.52 [5.41, 5.63]	5.50 [5.37, 5.63]	6.17 [6.02, 6.32]	4.92 [4.78, 5.05]	7.16 [7.00, 7.32]	7.23 [7.08, 7.39]
SUPERT	0.511 [0.509, 0.514]	0.536 [0.533, 0.539]	0.551 [0.548, 0.554]	0.548 [0.545, 0.551]	**0.557 * [0.554, 0.560]**	0.550 [0.547, 0.553]	**0.554 † [0.551, 0.557]**	0.553 [0.551, 0.556]	0.512 [0.510, 0.514]	0.521 [0.519, 0.523]	0.506 [0.504, 0.509]	0.504 [0.502, 0.506]
Stats-coverage	0.62 [0.62, 0.63]	0.66 [0.66, 0.66]	0.70 [0.69, 0.70]	0.69 [0.69, 0.70]	**0.72 * [0.72, 0.72]**	0.70 [0.69, 0.70]	0.71 [0.71, 0.72]	0.71 [0.71, 0.72]	0.56 [0.56, 0.56]	0.57 [0.56, 0.57]	0.54 [0.54, 0.54]	0.54 [0.53, 0.54]
SummaQA	0.063 [0.055, 0.071]	0.089 [0.079, 0.099]	**0.168 † [0.151, 0.184]**	0.156 [0.141, 0.172]	**0.180 * [0.164, 0.196]**	0.129 [0.117, 0.142]	**0.168 † [0.150, 0.187]**	**0.166 † [0.151, 0.181]**	0.055 [0.048, 0.062]	0.052 [0.044, 0.060]	0.043 [0.036, 0.050]	0.038 [0.033, 0.044]

Note that data are shown as mean [2.5th percentile, 97.5th percentile]. "*" denotes the highest value for each metric, and "†" denotes the values that do not have statistically significant difference (P>0.05) with the highest value.

Figure E3: Assessment of 12 language models using all evaluation metrics included in this study. Displayed numbers are actual metric values, and the 95% confidence intervals were determined via bootstrap resampling.

Appendix S8: Findings and Background Information for the Examples in Expert Evaluation

Figures E4, E5, E6 and E7 show the findings and background sections associated with Cases 1, 2, 3, 4, in Figure 5 (in the main body).

Indication: [AGE]-year-old [SEX] with pulmonary nodule, presents for a staging FDG PET/CT examination.

Findings:
Background liver metabolic activity (SUV mean/ SUV max): 3.9/5.7 (PET/CT axial slice 155).
Background mediastinal blood pool metabolic activity (SUV mean/ SUV max): 3.1/3.9 (PET/CT axial slice 119).
Head/Neck: No FDG avid cervical nodes are noted. Physiologic symmetric FDG uptake is present in the visualized portions of the brain, extraocular muscles, and salivary glands with no distinct focal abnormalities.
Chest: Redemonstration of a subpleural oval-shaped solid nodule within the anteroinferior right upper lobe immediately superior to the right minor fissure, measuring approximately 1.2 x 1.3 cm in size, unchanged compared to [DATE]. This has mild associated FDG uptake (SUV max 1.8, axial slice 125). With reference to outside prior CT, there is suggestion of macroscopic fat within the nodule. No other pulmonary nodules are identified. No pleural effusion.
No FDG avid lymph nodes are noted in the axillae, hila, or mediastinum. Physiologic FDG uptake is present within the myocardium. No pericardial effusion. Mild calcified atherosclerotic plaque along the aortic arch and at the origins of the great vessels.
Redemonstration of a mildly lobulated nodular area of soft tissue within the subareolar right breast. This measures approximately 1.2 x 1.1 cm in size, unchanged compared to [DATE]. This does not appear to have associated FDG uptake.
Abdomen/Pelvis: No FDG avid nodes or mesenteric lesions are noted. Heterogeneous FDG uptake is noted in the liver and spleen without focal abnormalities. The adrenal glands appear unremarkable. Mild uptake is noted along the bowel within the normal physiologic variation. Excreted radiotracer is present within the urinary collecting system and bladder. No hydronephrosis. Normal caliber abdominal aorta, with moderate calcified atherosclerotic plaque.
Musculoskeletal/Extremities:
No FDG avid skeletal lesions are noted. Infiltrated radiotracer is noted within the skeletal right upper arm antecubital region. There are no other areas of abnormal hypermetabolism within the visualized portions of the extremities.

Original clinical impression
[1] FDG PET/CT demonstrates short-term stability of the solitary nodule within the right upper lobe, which has only minimal associated FDG uptake, not elevated above mediastinal blood pool. Given the lack of significant FDG avidity, and the suggestion of macroscopic fat within the nodule on prior diagnostic chest CT, a benign lesion such as a pulmonary hamartoma is a consideration. Could consider short-term follow-up diagnostic CT chest in 3-6 months to assess for interval change.
[2] No evidence of suspected FDG avid local nodal mediastinal/hilar or distant metastatic disease.
[3] Mildly lobulated nodular soft tissue in the subareolar right breast, without significant FDG avidity. Could correlate with prior mammography, if available. If not, screening mammography is recommended.

PEGASUS-generated impression
[1] FDG PET/CT demonstrates a 1.2 x 1.3 cm subpleural solid nodule within the anteroinferior right upper lobe with mild associated FDG uptake, which is nonspecific by PET criteria for an infectious/inflammatory process versus low-grade primary lung malignancy. Recommend correlation with prior and/or follow-up thoracic CT to assess for interval change in size.
[2] No evidence of FDG avid local nodal mediastinal/hilar or distant metastatic disease.
[3] Redemonstration of a mildly lobulated nodular area of soft tissue within the subareolar right breast, unchanged compared to [DATE]. This does not appear to have associated hypermetabolic activity.

Figure E4: The findings section and relevant background information for Case 1 in Figure 5 (in the main body).

Indication: [AGE] years old patient with history of mantle cell lymphoma diagnosed by left axillary lymph node biopsy. Status post 3 cycles chemotherapy. Patient also has history of right breast lobular carcinoma in situ and ALH in [DATE] status post partial mastectomy and tamoxifen x5 years. Patient is referred for assessment of response to treatment.

Findings:
Physiologic background liver standardized uptake value (SUV mean and SUV max) reported for comparison between PET studies: 2.0 and 2.5, previously 2.3 and 2.9.
Visualized head/neck: Physiologic uptake in the visualized portions of the brain, extraocular muscles, and salivary glands. Decreased homogeneous uptake in the thyroid, right thyroid lobe SUV max 2.2, previously SUV max 5.0.
Head/neck lymph nodes: Interval resolution of previously noted hypermetabolic bilateral cervical lymphadenopathy. Currently there are few scattered subcentimeter lymph nodes that are not avid. No new suspicious head/neck lymph nodes.
Lungs: No lung nodules or abnormal uptake. Mild dependent atelectasis.
Pleura/pericardium: No pleural or pericardial effusion.
Thoracic lymph nodes: Decreased size and resolved uptake of mediastinal and axillary lymph nodes. For example:
-Left axillary lymph node 0.4 x 0.6 cm SUV max 0.8 (axial PET/CT slice 83), previously 1.2 x 0.8 cm SUV max 5.9
-Retrocrural lymph node 0.9 x 0.3 cm SUV max 1.6 (axial PET/CT slice 124), previously 1.5 x 0.7 cm SUV max 5.2.
Other chest findings: Physiologic myocardial uptake. Prior right breast lumpectomy.
Hepatobiliary: No abnormal uptake.
Spleen: Resolved splenomegaly and uptake measuring 9.8 cm in AP dimension SUV max 2.1, previously 22.1 cm SUV max 9.9.
Pancreas: No abnormal uptake.
Adrenals: Within normal limits.
Kidneys/bladder: No abnormal uptake. Physiologically excreted tracer activity within the renal collecting system and urinary bladder.
Bowel/peritoneum: No suspicious bowel uptake or abnormality. Diverticulosis without evidence of diverticulitis.
Pelvic organs: No abnormal uptake. Uterus is surgically absent.
Abdominopelvic lymph nodes: Decreased size and resolved uptake of multiple abdominopelvic lymph nodes. No new suspicious lymph nodes.
Musculoskeletal/soft tissues/skin: Decreased heterogeneous marrow uptake. A focus of relatively increased uptake at the T12 endplate without likely corresponds to degenerative changes. No suspicious osseous lesions.
Other: None.

Original clinical impression
[1] FDG PET/CT demonstrates interval resolution of multiple bilateral head/neck, thoracic, and abdominopelvic lymph nodes as well as resolved splenomegaly and intense splenic uptake consistent with complete metabolic response. Deauville 1.
[2] No new sites of lymphomatous involvement.

PEGASUS-generated impression
[1] FDG PET/CT demonstrates interval resolution of previously noted hypermetabolic lymphadenopathy in the neck, chest, abdomen, and pelvis consistent with complete metabolic response (Deauville score 1).
[2] Decreased homogeneous uptake in the thyroid.

Figure E5: The findings section and relevant background information for Case 2 in Figure 5 (in the main body).

Indication: Patient is a [AGE]-year-old [SEX] with stage IIIB Merkle cell carcinoma of the left eyebrow status post wide local excision with split-thickness skin graft, left superficial parotidectomy with facial nerve dissection and selective neck dissection levels 2A and 2B on [DATE]. This was followed by adjuvant radiation to the parotid bed, periparotid nodes, and cervical levels 2-4 completed in [DATE]. The purpose of the study is restaging of the disease.

Findings:
Mediastinal blood pool demonstrates mean SUV of 2.2 measured within the descending thoracic aorta at the level of the carina (axial PET/CT image 140); previously 2.3. Background liver demonstrates mean SUV of 2.6 measured within the inferior right hepatic lobe (axial PET/CT image 208); previously 2.7.
Head/neck: Note is made of slight interval increase in size and FDG uptake of a mildly hypermetabolic subcutaneous soft tissue nodule within the left neck anterior to the sternocleidomastoid muscle at the level of the thyroid cartilage. It appears more rounded and discrete on the current exam measuring approximately 1.2 cm with SUV max of 2.3 (axial PET/CT image 105) compared with previously 0.9 cm with SUV max of 1.6. Symmetric FDG uptake is present in the visualized portions of the brain, extraocular muscles, larynx, and salivary glands with no distinct focal abnormalities. No new or enlarging FDG-avid cervical lymphadenopathy is noted. Postsurgical changes of left neck dissection are stable with no evidence of suspicious FDG uptake. Oral cavity, oropharynx, nasopharynx, and larynx appear unremarkable. Thyroid gland is diminutive in appearance which is compatible with patient's history of hypothyroidism. Parotid and submandibular glands are unremarkable. Paranasal sinuses are well-aerated. Mastoid air cells and tympanic cavities are clear. No significant dental abnormalities are noted.
Chest: No new or enlarging FDG-nodules. No pleural effusion or pneumothorax. Central airways are widely patent. No new or enlarging FDG-avid axillary, mediastinal, or hilar lymphadenopathy is noted. Heart is mildly enlarged in size. Physiological FDG uptake is present within the myocardium. No pericardial effusion. Thoracic aorta is normal in course and caliber. Mild atheromatous calcifications are present in the thoracic aorta and left anterior descending coronary artery.
Abdomen/Pelvis: Expected physiologic FDG uptake is noted within the solid and hollow abdominopelvic viscera. Non-FDG avid high-attenuation cysts in both kidneys are stable and may be proteinaceous or hemorrhagic in nature. Representative lesion at the superior pole of the left kidney measures 2.0 cm in size and 1.5 cm upper pole right kidney. Photopenic 5-mm simple cyst in the interpolar region of the right kidney. Tiny nonobstructing calculus is noted in the right kidney. There is sigmoid diverticulosis without CT-evidence of diverticulitis. Surgical absence of the uterus. Excreted radiotracer is present within the urinary collecting system and bladder. Right ureter is mildly prominent unchanged from before. No FDG-avid abdominopelvic lymphadenopathy is noted. Atherosclerotic calcifications are present in the nonaneurysmal abdominal aorta and iliac arteries.
Musculoskeletal: No suspicious FDG uptake is noted in the region of the left eyebrow when compared to PET/PET dated [DATE]. No suspicious FDG uptake is noted elsewhere in the skin or muscle or bone. Degenerative disc and facet disease is noted in the spine with diffuse demineralization. Superficial venous collaterals are noted in bilateral lower extremities.

Original clinical impression
[1] Slight interval increase in size and FDG uptake of a mildly hypermetabolic subcutaneous soft tissue nodule in the left neck anterior to the sternocleidomastoid muscle which appears more rounded and discrete on the current exam when compared to most recent PET/CT from [DATE]. This finding possibly represents metastatic disease but not particularly avid. Recommend ultrasound-guided sampling for biopsy confirmation.
[2] No abnormal FDG uptake to suggest FDG-avid locally recurrent or additional sites of metastatic disease.
[3] Probable proteinaceous/hemmorhagic renal cysts. Recommend confirmation with ultrasound.

PEGASUS-generated impression
[1] Slight interval increase in size and FDG uptake of a mildly hypermetabolic subcutaneous soft tissue nodule within the left neck anterior to the sternocleidomastoid muscle at the level of the thyroid cartilage is favored to represent post therapeutic inflammation rather than recurrent disease. Recommend attention on follow-up.
[2] No evidence of FDG-avid local or distant metastatic disease.

Figure E6: The findings section and relevant background information for Case 3 in Figure 5 (in the main body).

Indication: [AGE]-year-old [SEX] with history of invasive ductal carcinoma of the left breast. Left axillary lymphadenopathy seen on initial staging. Also with concern for osseous metastatic disease in the pelvis, left clavicle, and lumbar spine. At the end of 2018 progressive disease was seen in the axilla. Currently treated with Fulvestrant (with Palbociclib) and Zometa. Most recent imaging with some suspicious nodular tissue posterior to the left breast clip as well as enlarging left axillary lymphadenopathy ([DATE]). Request to evaluate for disease status.

Findings:
Background liver metabolic activity (SUV mean/ SUV max): 3.0/3.9 (PET/CT axial slice 158); Background mediastinal blood pool metabolic activity (SUV mean/ SUV max): 2.4/2.7 (PET/CT axial slice 113);
Skull base/Neck: No FDG avid cervical nodes are noted. There is moderate FDG activity associated with the eyelids bilaterally. Additionally there is some mild-moderate activity associated with the nasal mucosa which may represent mild nonspecific inflammation. Physiologic symmetric FDG uptake is present in the visualized portions of the brain, extraocular muscles, and salivary glands with no distinct focal abnormalities. Likely meningioma near the falx in the right frontal region. Paranasal sinuses are free of significant disease. Tympanic and mastoid air cells clear.
Chest: Redemonstration of left axillary lymphadenopathy which demonstrates moderate-intense FDG avidity. Overall these appear similar in size and distribution compared to [DATE]. For example a posterior axillary lymph node measures 11 mm (PET/CT axial slice 109; SUV max 13.2) compared to 11 mm previously. A lower axillary lymph node measures 13 mm (PET/CT/CT axial slice 122; SUV max 6.0) compared to 14 mm previously. The area of nodular soft tissue at the posterior aspect of the left breast glandular tissue, near the biopsy clip, which appear to be enlarging on previous CT examinations demonstrates intense FDG avidity (SUV max 13.8; PET/CT axial slice 134). The area of FDG avidity measures approximately 2.7 x 3.5 x 3.9 cm (LR-AP-CC) in maximal dimension. No FDG avid lung nodules are noted. Physiologic FDG uptake is present within the myocardium. Unchanged heart size. No pericardial or pleural effusion. No pneumothorax. Dependent atelectasis. Calcified hilar and subcarinal lymphadenopathy suggestive of prior granulomatous infection.
Abdomen/Pelvis: There is slight misregistration, especially in the upper abdomen, due to patient motion.
No FDG avid nodes or mesenteric lesions are noted. Heterogeneous FDG uptake is noted in the liver and spleen without focal abnormalities. The adrenal glands appear unremarkable. Moderate uptake is noted along the bowel. There is no corresponding focal CT abnormality seen. Excreted radiotracer is present within the urinary collecting system and bladder. The unenhanced contours of the liver, spleen, adrenal glands, pancreas are within normal limits. The gallbladder is surgically absent. Unchanged mild dilatation of the extrahepatic bile ducts, consistent with reservoir effect. Symmetric renal cortical thickness. No hydronephrosis. No bowel obstruction. Scattered colonic diverticula without CT findings of diverticulitis. Appendix surgically absent. No adnexal masses. No lymphadenopathy in the abdomen or pelvis.
Musculoskeletal/Extremities: There is heterogeneous mild FDG activity associated with the osseous pelvis which corresponds to a very mottled sclerotic and lytic appearance. Within the T8 vertebral body there is a small focus of increased FDG avidity (SUV max 3.3; PET/CT axial slice 129) which does not have a clear CT correlate. There is also some heterogeneous mildly increased FDG uptake associated with the lumbar vertebral bodies, nonspecific. There is no definite focal increased FDG uptake associated with the left clavicle which was the site of previously suspected metastatic disease. Elsewhere there is increased uptake associated with the bilateral shoulders, left C2/C3 facet joint, additional intervertebral disk and facet spaces, and the bilateral hips most suggestive of degenerative change.

Original clinical impression
[1] FDG PET/CT demonstrates intense FDG activity associated with the area of nodular soft tissue thickening at the posterior aspect of the left breast, highly suspicious for recurrent/residual malignancy.
[2] Intense FDG avidity associated with the left axillary lymphadenopathy, which is overall unchanged in size and distribution compared to the recent [DATE] CT, also highly suspicious for persistent nodal metastatic disease.
[3] No definite evidence of FDG avid osseous metastatic disease. Mild heterogeneous FDG activity, similar to normal bone marrow activity, corresponding to mottled sclerotic and lytic changes in the pelvis, lumbar spine and T8 vertebral body, most likely represents physiologic bone marrow activity at sites of previously treated osseous metastatic disease.

PEGASUS-generated impression
[1] FDG PET/CT demonstrates intense FDG activity associated with the enlarging nodular soft tissue at the posterior aspect of the left breast glandular tissue, which appears to be enlarging on previous CT examinations. This finding is highly suspicious for recurrent malignancy.
[2] Moderate-intense FDG avid left axillary lymphadenopathy, similar in size and distribution compared to recent chest CT from [DATE], compatible with nodal metastatic disease.
[3] Heterogeneous mild to moderate FDG uptake associated with sclerotic and lytic osseous changes in the pelvis, left clavicle, and T8 vertebral body, with no definite correlative CT bone abnormality on our corresponding low-dose noncontrast CT. These findings are nonspecific but favored to represent posttreatment related inflammatory change rather than residual/recurrent disease. Recommend attention to these sites on follow-up imaging.
[4] No evidence of FDG-avid distant metastatic disease in the chest, abdomen, or pelvis.

Figure E7: The findings section and relevant background information for Case 4 in Figure 5 (in the main body).

Appendix S9: Findings and Background Information for the Examples in Encoding Physician-specific Styles

Figures E8 and E9 show the findings and background sections associated with Cases 1 and 2 in Figure 6 (in the main body).

Indication: [AGE]-year-old [SEX] with recently diagnosed poorly adenocarcinoma of the right upper lobe status post biopsy on [DATE]. Patient is referred for initial staging.

Findings:
Physiologic background liver standardized uptake value (SUV mean and SUV max) reported for comparison between PET studies: 2.7 and 3.8.
Visualized head/neck: Physiologic uptake in the visualized portions of the brain, extraocular muscles, and salivary glands.
Head/neck lymph nodes: No suspicious head/neck lymph nodes.
Lungs: Medial right upper lobe apical segment pulmonary nodule abutting the pleura measuring 1.5 x 1.6 cm, SUV max 13.5, axial image 70. No additional nodules. Right middle lobe granuloma. Left lingular atelectasis/scarring.
Pleura/pericardium: Mild to moderate FDG activity corresponding same right lower lobe posterior pleural thickening at the 8th/9th intercostal region measuring 0.8 x 0.4 cm, SUV max 3.7, axial image 110, suspicious for a pleural metastatic implant. This focus appears to correspond to subtle oral thickening seen on chest CT from [DATE] (series 3, axial slice 315; series 2, axial slice 79). This focus does not appear to be misregistered PET activity adjacent lung or bone to suggest FDG avid osseous or additional lung metastasis. This focus is less likely to represent top normal physiologic muscle activity. No pleural or pericardial effusion.
Thoracic lymph nodes: No suspicious thoracic lymph nodes. Scattered calcified hilar and mediastinal lymph nodes.
Other chest findings: Physiologic myocardial uptake. Moderate coronary artery calcifications.
Hepatobiliary: No abnormal uptake. Spleen: No abnormal uptake. Pancreas: No abnormal uptake. Adrenals: Non-FDG avid left adrenal gland nodule measuring 1.3 cm, SUV max 2.2, with fat attenuation on noncontrast CT (-18 Hounsfield units). Normal right adrenal gland. Kidneys/bladder: No abnormal uptake. Physiologically excreted tracer activity within the renal collecting system and urinary bladder. Exophytic right and left renal cysts. Bowel/peritoneum: No suspicious bowel uptake or abnormality. Pelvic organs: No abnormal uptake. Abdominopelvic lymph nodes: No suspicious abdominopelvic lymph nodes.
Musculoskeletal/soft tissues/skin: Degenerative changes of the spine. No suspicious osseous lesion.
Other: None.

Original clinical impression dictated by Physician 1	PEGASUS-generated impression in Physician 1's style	PEGASUS-generated impression in Physician 2's style
[1] FDG PET/CT demonstrates an intensely FDG medial right upper lobe apical pulmonary nodule abutting the pleura, compatible with biopsy-proven primary lung malignancy.<br>[2] Mild to moderate focus of FDG activity in the posterior right lower lobe corresponding to subtle soft tissue pleural thickening at the 8th/9th intercostal region on CT, suspicious for a metastatic pleural implant.<br>[3] No evidence of other sites of FDG avid local nodal or distant metastatic disease.<br>[4] Stable non-FDG left adrenal gland nodule dating to [DATE], most consistent with a benign myolipoma..	[1] FDG PET/CT demonstrates an intensely FDG avid 1.5 x 1.6 cm right upper lobe apical segment pulmonary nodule, consistent with biopsy-proven primary lung malignancy.<br>[2] Mild to moderate FDG activity corresponding right lower lobe posterior pleural thickening at the 8th/9th intercostal region, suspicious for a pleural metastatic implant.<br>[3] No evidence of other sites of suspected PET avid local nodal or distant metastatic disease.<br>[4] Non-FDG avid 1.3 cm left adrenal nodule, with fat attenuation on noncontrast CT, which is indeterminate. Recommend adrenal protocol CT or MRI for further evaluation.	[1] Right upper lobe primary lung malignancy with suspected right lower lobe pleural metastasis.<br>[2] No evidence of nodal or distant metastases.<br>[3] Indeterminate left adrenal nodule can be followed on future surveillance imaging.

Figure E8: The findings section and relevant background information for Case 1 in Figure 6 (in the main body).

Indication: [AGE] year old [SEX] with new diagnosis of right upper lobe nodule biopsy consistent with squamous cell carcinoma. Patient is referred for initial staging.

Findings:
Physiologic background liver standardized uptake value (SUV mean and SUV max) reported for comparison between PET studies: 2.6 and 5.0.
Visualized head/neck: Physiologic uptake in the visualized portions of the brain, extraocular muscles, and salivary glands.
Head/neck lymph nodes: No suspicious head/neck lymph nodes.
Lungs: Avid right upper lobe mass is consistent with biopsy-proven primary lung malignancy, 3.0 x 2.4 cm, SUV max 7.5 (series 1200 image 89). Other lung findings are better evaluated on recent dedicated chest CT. No other abnormal uptake. Emphysema.
Pleura/pericardium: No pleural or pericardial effusion. Thoracic lymph nodes: No suspicious thoracic lymph nodes. Uptake of the mediastinal lymph nodes is probably reactive/inflammatory. Other chest findings: Coronary vascular and scattered thoracic aortic calcification. Bilateral gynecomastia. Benign incidental tracheal diverticulum.
Hepatobiliary: No abnormal uptake. Prior liver transplant. Fatty liver. Cholecystectomy. Spleen: No abnormal uptake. Pancreas: No abnormal uptake. Adrenals: Within normal limits. Kidneys/bladder: No abnormal uptake. Physiologically excreted tracer activity within the renal collecting system and urinary bladder. Bowel/peritoneum: No suspicious bowel uptake or abnormality. Diverticulosis without inflammatory change. Pelvic organs: No abnormal uptake. Abdominopelvic lymph nodes: No suspicious abdominopelvic lymph nodes.
Musculoskeletal/soft tissues/skin: No suspicious osseous lesions. Diffuse marrow uptake is probably reactive and benign. Multilevel spine DJD. Prior lumbar laminectomy. Reactive uptake near the left greater trochanter.
Other: Aortoiliac atherosclerotic vascular calcifications. Small umbilical hernia."

Original clinical impression dictated by Physician 2	PEGASUS-generated impression in Physician 1's style	PEGASUS-generated impression in Physician 2's style
Primary right upper lobe lung malignancy without evidence for nodal or distant metastases.	[1] FDG PET/CT demonstrates an avid right upper lobe mass consistent with biopsy-proven primary lung malignancy.<br>[2] No evidence of other sites of abnormal FDG uptake to suggest locoregional or distant metastatic disease.	Right upper lobe primary lung malignancy without evidence of nodal or distant metastases.

Figure E9: The findings section and relevant background information for Case 2 in Figure 6 (in the main body).

Appendix S10: External Testing

Table E3 presents the performance of PEGASUS in the external test set, assessed using automatic evaluation metrics. The first row shows the results of internal testing, while the following three rows display the external test results given impressions generated in the styles of Physician 1, 2, and 3, respectively. Figure E10 provides 4 sample cases with original clinical impressions dictated by different physicians in the external set.

Table E3: Performance of PEGASUS in the external test set.

	BARTScore +PET	PEGASUSScore +PET	ROUGE-1	ROUGE-2	ROUGE-L	BLEU	BERTScore
**Internal test**	-1.47 [-1.48, -1.46]	-1.44 [-1.45, -1.42]	53.8 [53.4, 54.2]	30.9 [30.5, 31.4]	40.0 [39.6, 40.5]	24.7 [24.2, 25.1]	0.747 [0.735, 0.739]
**External test using Physician 1's style**	-1.66 [-1.70, -1.62]	-1.72 [-1.77, -1.67]	38.6 [36.9, 40.2]	14.8 [13.5, 16.1]	26.2 [24.9, 27.6]	11.1 [9.9, 12.3]	0.671 [0.662, 0.679]
**External test using Physician 2's style**	-1.68 [-1.73, -1.63]	-1.67 [-1.72, -1.61]	38.5 [36.5, 40.5]	15.9 [14.1, 17.8]	29.2 [27.2, 31.3]	11.5 [9.8, 13.4]	0.679 [0.668, 0.691]
**External test using Physician 3's style**	-1.73 [-1.78, -1.68]	-1.75 [-1.81, -1.69]	42.2 [40.6, 43.8]	18.1 [16.5, 19.7]	30.0 [28.4, 31.8]	13.3 [11.8, 14.9]	0.688 [0.679, 0.697]

Note that a higher value indicates better performance for all these metrics. We picked BARTScore+PET and PEGASUSScore+PET, as they are most correlated with physician preferences. We also included the results of ROUGE, BLEU and BERTScore because they are commonly used metrics in radiology report summarization. Data are shown as mean [2.5th percentile, 97.5th percentile].

	**Original clinical impression in the external test set**	**PEGASUS-generated impression in Physician 1's style**	**PEGASUS-generated impression in Physician 2's style**	**PEGASUS-generated impression in Physician 3's style**
1	[1] Large metabolically active anterior mediastinal mass with metabolically active left supraclavicular and left axillary lymphadenopathy as described above. There is also a small focus of increased uptake in the anterior mediastinum to the right of the large mass, which could represent another small lymph node.<br>[2] Metabolically active focus of ground-glass opacity posteriorly in the left lower lobe. This may be inflammatory in nature, and continued follow-up with attention to this lesion is recommended.<br>[3] Diffuse bone marrow uptake in the axial skeleton. Correlation with bone marrow biopsy is recommended.	[1] FDG PET/CT demonstrates a large anterior mediastinal mass with intense FDG uptake consistent with the patient's biopsy-proven Hodgkin's lymphoma. There is also FDG avid left supraclavicular and left axillary lymphadenopathy.<br>[2] There is a small focus of increased uptake in the anterior mediastinum to the right of the large mass.<br>[3] There is an approximately 8.4 mm area of ground-glass opacity posteriorly in the left lower lobe that is metabolically active with a maximal SUV of 3.8. This finding is nonspecific and may represent an infectious/inflammatory process although a site of metastatic disease cannot be completely excluded. Recommend correlation with prior and/or follow-up thoracic CT to assess for interval change in size.<br>[4] There is diffuse FDG activity in the bone marrow of the axial skeleton, likely reactive hematopoiesis.	[1] Large anterior mediastinal mass with left supraclavicular and left axillary lymphadenopathy that is metabolically active consistent with the provided history of Hodgkin's lymphoma.<br>[2] A small focus of uptake in the anterior mediastinum to the right of the large mass is suspicious for lymphomatous involvement.<br>[3] Ground-glass opacity in the left lower lobe with mild uptake is indeterminate and can be followed on future surveillance imaging.<br>[4] Diffuse uptake of the bone marrow is probably reactive.	[1] Large anterior mediastinal mass with associated metabolically active left supraclavicular and left axillary lymphadenopathy. There is also a small focus of increased uptake in the anterior mediastinum to the right of the large mass.<br>[2] There is an approximately 8.4 mm area of ground-glass opacity posteriorly in the left lower lobe with a maximal SUV of 3.8. This might represent an inflammatory focus, however metastatic disease cannot be excluded.<br>[3] Diffuse uptake is identified in the bone marrow of the axial skeleton. This likely represents reactive bone marrow.
2	Abnormal study. Metabolically active nodal disease above and below the diaphragm, with extranodal hypermetabolic involvement of the spleen, correlates with the histologically proven Hodgkin's lymphoma.	[1] FDG PET/CT demonstrates extensive intensely hypermetabolic lymphadenopathy throughout the neck, chest, abdomen, and pelvis, as detailed in the findings, consistent with biopsy-proven lymphoma.<br>[2] Diffusely increased metabolic activity in the spleen is nonspecific and may represent lymphomatous involvement versus physiologic splenic activity.<br>[3] No evidence of FDG avid bone marrow or osseous involvement.	[1] Bulky, intensely hypermetabolic lymphadenopathy in the neck, chest, abdomen, and pelvis, consistent with known diagnosis of Hodgkin lymphoma.<br>[2] Diffusely increased metabolic activity in the spleen is also suspicious for lymphoma involvement.	[1] Deauville score 5.<br>[2] Bulky, intensely hypermetabolic lymphadenopathy in the neck, chest, abdomen, and pelvis, consistent with biopsy-proven diagnosis of Hodgkin's lymphoma.<br>[3] Diffusely increased metabolic activity in the spleen is also concerning for lymphomatous involvement.
3	Hypermetabolic adenopathy consistent with newly diagnosed Hodgkin's lymphoma in neck, chest, abdomen, and possibly in pelvis, as noted above.	[1] FDG PET/CT demonstrates hypermetabolic adenopathy in the neck, chest, abdomen, and pelvis, as detailed in the findings, consistent with Hodgkin's lymphoma. Deauville score 5.<br>[2] Hypermetabolic splenic masses are also consistent with lymphomatous involvement.<br>[3] Diffuse increased marrow activity which could be secondary to anemia. Recommend clinical correlation.	Hypermetabolic adenopathy in the neck, chest, abdomen, and pelvis is consistent with lymphoma (Deauville 5).	[1] Hypermetabolic adenopathy in the neck, chest, abdomen, and pelvis, as detailed above, consistent with lymphoma. Deauville score 5.<br>[2] Diffuse increased marrow activity, likely secondary to anemia.
4	[1] Continued interval decrease in size and/or metabolic activity of lymph nodes in the neck and mediastinum, consistent with continued therapy. Faint radiotracer activity remaining in the mediastinal lymph nodes is less than or equal to that seen in the mediastinal blood pool. This would be consistent with a Deauville score 2.<br>[2] Interval decrease in size and metabolic activity of the previously seen right middle lobe nodule, consistent with resolving inflammation or infection. Resolving, treated malignancy could also have this appearance.<br>[3] No new foci of increased radiotracer activity are identified to suggest new metastatic disease.	[1] FDG PET/CT demonstrates further interval decrease in size and metabolic activity of the previously seen cervical and mediastinal lymphadenopathy, consistent with response to interval therapy. Deauville score 2.<br>[2] Interval resolution of previously seen hypermetabolic right middle lobe pulmonary nodule.<br>[3] No evidence of new sites of FDG avid malignancy.<br>[4] Mild diffuse marrow activity throughout the axial skeleton consistent with recent chemotherapy.	[1] Further decrease in size and metabolic activity of the cervical and mediastinal lymph nodes, consistent with response to treatment.<br>[2] Decreased size of the right middle lobe pulmonary nodule.<br>[3] No evidence of new disease.	[1] Further decrease in size and metabolic activity of the previously seen cervical and mediastinal lymph nodes. No new hypermetabolic lymph nodes are identified. Deauville score 2.<br>[2] Interval decrease in metabolic activity and size of the right middle lobe pulmonary nodule.<br>[3] Mild diffuse marrow activity throughout the axial skeleton, consistent with marrow hyperplasia from recent chemotherapy.

Figure E10: Examples of PEGASUS-generated impressions for the external whole-body PET/CT reports. The first column shows the reference clinical impressions. Subsequent columns present impressions generated in the styles of Physician 1, 2, and 3 from our internal dataset.

References

1. Zhang Y, Ding DY, Qian T, Manning CD, Langlotz CP. Learning to Summarize Radiology Findings. Proc Ninth Int Workshop Health Text Min Inf Anal. Brussels, Belgium: Association for Computational Linguistics; 2018. p. 204–213. doi: http://doi.org/10.18653/v1/W18-5623.
2. Chen C, Yin Y, Shang L, et al. bert2BERT: Towards Reusable Pretrained Language Models. Proc 60th Annu Meet Assoc Comput Linguist Vol 1 Long Pap. Dublin, Ireland: Association for Computational Linguistics; 2022. p. 2134–2148. doi: http://doi.org/10.18653/v1/2022.acl-long.151.
3. Li Y, Wehbe RM, Ahmad FS, Wang H, Luo Y. Clinical-Longformer and Clinical-BigBird: Transformers for long clinical sequences. arXiv; 2022. http://arxiv.org/abs/2201.11838. Accessed August 16, 2023.
4. Liu Y, Ott M, Goyal N, et al. RoBERTa: A Robustly Optimized BERT Pretraining Approach. arXiv; 2019. http://arxiv.org/abs/1907.11692. Accessed August 16, 2023.
5. Lewis M, Liu Y, Goyal N, et al. BART: Denoising Sequence-to-Sequence Pre-training for Natural Language Generation, Translation, and Comprehension. arXiv; 2019. http://arxiv.org/abs/1910.13461. Accessed March 7, 2023.
6. Yuan H, Yuan Z, Gan R, Zhang J, Xie Y, Yu S. BioBART: Pretraining and Evaluation of A Biomedical Generative Language Model. arXiv; 2022. http://arxiv.org/abs/2204.03905. Accessed August 15, 2023.
7. Zhang J, Zhao Y, Saleh M, Liu PJ. PEGASUS: Pre-training with Extracted Gap-sentences for Abstractive Summarization. arXiv; 2020. http://arxiv.org/abs/1912.08777. Accessed March 7, 2023.
8. Raffel C, Shazeer N, Roberts A, et al. Exploring the Limits of Transfer Learning with a Unified Text-to-Text Transformer. arXiv; 2020. http://arxiv.org/abs/1910.10683. Accessed August 14, 2023.
9. Lu Q, Dou D, Nguyen TH. ClinicalT5: A Generative Language Model for Clinical Text. Findings of the Association for Computational Linguistics: EMNLP 2022, pages 5436–5443, Abu Dhabi, United Arab Emirates. Association for Computational Linguistics. doi: http://doi.org/10.18653/v1/2022.findings-emnlp.398.
10. Johnson AEW, Pollard TJ, Berkowitz SJ, et al. MIMIC-CXR, a de-identified publicly available database of chest radiographs with free-text reports. Sci Data. 2019;6(1):317. doi: http://doi.org/10.1038/s41597-019-0322-0.
11. Wei J, Bosma M, Zhao VY, et al. Finetuned Language Models Are Zero-Shot Learners. arXiv; 2022. http://arxiv.org/abs/2109.01652. Accessed August 15, 2023.
12. Ziegler DM, Stiennon N, Wu J, et al. Fine-Tuning Language Models from Human Preferences. arXiv; 2020. http://arxiv.org/abs/1909.08593. Accessed August 14, 2023.
13. Zhang S, Roller S, Goyal N, et al. OPT: Open Pre-trained Transformer Language Models. arXiv; 2022. http://arxiv.org/abs/2205.01068. Accessed February 22, 2023.
14. Touvron H, Lavril T, Izacard G, et al. LLaMA: Open and Efficient Foundation Language Models. arXiv; 2023. http://arxiv.org/abs/2302.13971. Accessed August 14, 2023.
15. Hu EJ, Shen Y, Wallis P, et al. LoRA: Low-Rank Adaptation of Large Language Models. arXiv; 2021. http://arxiv.org/abs/2106.09685. Accessed August 15, 2023.
16. Taori R, Gulrajani I, Zhang T, et al. Stanford Alpaca: An Instruction-following LLaMA model. GitHub; 2023. https://github.com/tatsu-lab/stanford_alpaca. Accessed June 20, 2023.
17. Loshchilov I, Hutter F. Decoupled Weight Decay Regularization. arXiv; 2019. http://arxiv.org/abs/1711.05101. Accessed August 31, 2023.
18. Lin CY. ROUGE: A package for automatic evaluation of summaries. In Text Summarization Branches Out, Barcelona, Spain, July 2004. Association for Computational Linguistics, 2004; 74–81. https://aclanthology.org/W04-1013/.
19. Papineni K, Roukos S, Ward T, Zhu W-J. BLEU: a method for automatic evaluation of machine translation. Proc 40th Annu Meet Assoc Comput Linguist - ACL 02. Philadelphia, Pennsylvania: Association for Computational Linguistics; 2001. p. 311. doi: http://doi.org/10.3115/1073083.1073135.
20. Popović M. chrF: character n-gram F-score for automatic MT evaluation. Proc Tenth Workshop Stat Mach Transl. Lisbon, Portugal: Association for Computational Linguistics; 2015. p. 392–395. doi: http://doi.org/10.18653/v1/W15-3049.
21. Banerjee, S. and Lavie, A. METEOR: An Automatic Metric for MT Evaluation with Improved Correlation with Human Judgments. Proceedings of Workshop on Intrinsic and Extrinsic Evaluation Measures for MT and/or Summarization. Ann Arbor, Michigan: Association of Computational Linguistics, 2005. p. 65–72.

22. Vedantam R, Zitnick CL, Parikh D. CIDEr: Consensus-based Image Description Evaluation. arXiv; 2015. http://arxiv.org/abs/1411.5726. Accessed August 31, 2023.
23. Ng J-P, Abrecht V. Better Summarization Evaluation with Word Embeddings for ROUGE. arXiv; 2015. http://arxiv.org/abs/1508.06034. Accessed August 31, 2023.
24. Zhang T, Kishore V, Wu F, Weinberger KQ, Artzi Y. BERTScore: Evaluating Text Generation with BERT. arXiv; 2020. http://arxiv.org/abs/1904.09675. Accessed August 22, 2023.
25. Zhao W, Peyrard M, Liu F, Gao Y, Meyer CM, Eger S. MoverScore: Text Generation Evaluating with Contextualized Embeddings and Earth Mover Distance. Proc 2019 Conf Empir Methods Nat Lang Process 9th Int Jt Conf Nat Lang Process EMNLP-IJCNLP. Hong Kong, China: Association for Computational Linguistics; 2019. p. 563–578. doi: http://doi.org/10.18653/v1/D19-1053.
26. Hu J, Li J, Chen Z, et al. Word Graph Guided Summarization for Radiology Findings. arXiv; 2021. http://arxiv.org/abs/2112.09925. Accessed March 2, 2023.
27. Yuan W, Neubig G, Liu P. BARTScore: Evaluating Generated Text as Text Generation. arXiv; 2021. http://arxiv.org/abs/2106.11520. Accessed August 15, 2023.
28. Thompson B, Post M. Automatic Machine Translation Evaluation in Many Languages via Zero-Shot Paraphrasing. Proc 2020 Conf Empir Methods Nat Lang Process EMNLP. Online: Association for Computational Linguistics; 2020. p. 90–121. doi: http://doi.org/10.18653/v1/2020.emnlp-main.8.
29. Peyrard M, Botschen T, Gurevych I. Learning to Score System Summaries for Better Content Selection Evaluation. Proc Workshop New Front Summ. Copenhagen, Denmark: Association for Computational Linguistics; 2017. p. 74–84. doi: http://doi.org/10.18653/v1/W17-4510.
30. Zhong M, Liu Y, Yin D, et al. Towards a Unified Multi-Dimensional Evaluator for Text Generation. Proc 2022 Conf Empir Methods Nat Lang Process. Abu Dhabi, United Arab Emirates: Association for Computational Linguistics; 2022. p. 2023–2038. doi: http://doi.org/10.18653/v1/2022.emnlp-main.131.
31. Scialom T, Lamprier S, Piwowarski B, Staiano J. Answers Unite! Unsupervised Metrics for Reinforced Summarization Models. arXiv; 2019. http://arxiv.org/abs/1909.01610. Accessed August 31, 2023.
32. Lita LV, Rogati M, Lavie A. BLANC: learning evaluation metrics for MT. Proc Conf Hum Lang Technol Empir Methods Nat Lang Process - HLT 05. Vancouver, British Columbia, Canada: Association for Computational Linguistics; 2005. p. 740–747. doi: http://doi.org/10.3115/1220575.1220668.
33. Gao Y, Zhao W, Eger S. SUPERT: Towards New Frontiers in Unsupervised Evaluation Metrics for Multi-Document Summarization. Proc 58th Annu Meet Assoc Comput Linguist. Online: Association for Computational Linguistics; 2020. p. 1347–1354. doi: http://doi.org/10.18653/v1/2020.acl-main.124.
34. Grusky M, Naaman M, Artzi Y. Newsroom: A Dataset of 1.3 Million Summaries with Diverse Extractive Strategies. Proc 2018 Conf North Am Chapter Assoc Comput Linguist Hum Lang Technol Vol 1 Long Pap. New Orleans, Louisiana: Association for Computational Linguistics; 2018. p. 708–719. doi: http://doi.org/10.18653/v1/N18-1065.
35. Fabbri AR, Kryściński W, McCann B, Xiong C, Socher R, Radev D. SummEval: Re-evaluating Summarization Evaluation. Trans Assoc Comput Linguist. 2021;9:391–409. doi: http://doi.org/10.1162/tacl_a_00373.
36. Huemann Z, Lee C, Hu J, Cho SY, Bradshaw T. Domain-adapted large language models for classifying nuclear medicine reports. arXiv; 2023. http://arxiv.org/abs/2303.01258. Accessed March 17, 2023.
